# Marital Conflict, Anger Expression, and Marital Instability: Associations by Age and Culture

**DOI:** 10.1093/geroni/igab046.2838

**Published:** 2021-12-17

**Authors:** Dakota Witzel, Madeline Nichols, Robert Stawski

**Affiliations:** Oregon State University, Corvallis, Oregon, United States

## Abstract

Positive social relationships, such as high-quality marriages, are associated with better health, especially among older adults. Moreover, negative components in marriages (i.e., disagreements and associated emotional responses) are linked to negative outcomes such as marital disruption (McGonagle et al., 1993) and divorce (Markman et al., 2010). Factors such as marital conflicts and emotion expression threaten marital stability and health and have been shown to decrease with age and in collectivist cultures (i.e., Japan compared to U.S.; Kitayama et al., 2015; Matsunaga & Imahori, 2009). While anger has featured as a specific emotion associated with compromised health and marital quality (Carrère et al., 2005), less is known about how marital conflict and the expression of anger may contribute to marital instability in later life, or differences in these links across age and culture. Using data from married respondents participating in the second wave of the Midlife in the United States (MIDUS, n=750, Mage=55.18, SD=11.5, %Female=50.13%) study or first wave of the Midlife in Japan (MIDJA; n=706, Mage=55.26, SD=13.68, %Female=47.73%) studies, we examined associations between disagreements, anger expression, and marital risk. Preliminary analyses revealed that marital disagreements and anger expression were each associated with increased marital instability in both the MIDUS and MIDJA samples (ps<.05). Additionally, the effects of marital disagreements and anger expression did not differ between the two samples or as a function of age. Discussion will focus on the relevance of expression and regulation of emotions for understanding marital (in)stability in midlife and aging and across cultures.

